# A novel *GIT2-BRAF* fusion in pilocytic astrocytoma

**DOI:** 10.1186/s13000-017-0669-5

**Published:** 2017-11-15

**Authors:** Jeffrey Helgager, Hart G. Lidov, Navin R. Mahadevan, Mark W. Kieran, Keith L. Ligon, Sanda Alexandrescu

**Affiliations:** 1 Department of Pathology, Boston Children’s Hospital, 300 Longwood Ave, Bader, Boston, MA 02115 USA; 2 Department of Pediatric Oncology, Dana-Farber Cancer Institute, 450 Brookline Ave, Boston, MA 02115 USA; 3 Department of Pathology, Brigham and Women’s Hospital, 55 Francis Street, Boston, MA 02115 USA

**Keywords:** Pilocytic astrocytoma, GIT2-BRAF, Fusion, BRAF

## Abstract

**Background:**

*KIAA1549-BRAF* fusion is the most common genetic event in pilocytic astrocytoma (PA), and leads to activation of the mitogen activated protein kinase (MAPK) signaling pathway. Fusions of *BRAF* with other partner genes, as well as other genetic alterations not involving BRAF but also leading to MAPK pathway activation have been described rarely.

**Case presentation:**

We present a new fusion partner in the low-grade glioma of a 10-year-old male, who presented with headaches and recent episodes of seizures. Magnetic resonance imaging (MRI) demonstrated a right temporal lobe tumor. Histological and immunohistochemical evaluation, and a next generation sequencing assay (Oncopanel, Illumina, 500 genes) including breaKmer analysis for chromosomal rearrangements were performed.

Histology was remarkable for a low-grade glioma composed of mildly atypical astrocytes with piloid processes, in a focally microcystic background. Mitoses were not seen; unequivocal Rosenthal fibers or eosinophilic granular bodies were absent. The tumor was positive for OLIG2 and GFAP and negative for BRAF V600E and IDH1 R132H mutant protein immunostains. Oncopanel showed low *SOX2* (3q26.33) copy number gain, and no gains at 7q34. There were no significant single nucleotide variants. BreaKmer detected a *GIT2-BRAF* fusion with loss of *BRAF* exons 1–8. The integrated diagnosis was low-grade glioma with piloid features, most consistent with pilocytic astrocytoma, WHO grade I.

**Conclusion:**

*GIT2-BRAF* fusion has not been reported in the literature in any tumor. Given that the *BRAF* sequence deleted is identical to that seen in other fusion events in PA, it most likely acts as tumor driver by activation of the MAPK pathway.

**Electronic supplementary material:**

The online version of this article (10.1186/s13000-017-0669-5) contains supplementary material, which is available to authorized users.

## Background

PA is the most common glioma in the pediatric population [1] and it represents 5.4% of all gliomas in children and adults [2]. These tumors can arise anywhere within the neuraxis, however have a predilection for the posterior fossa, most classically the cerebellum [3]. On imaging, PA is usually well-circumscribed, cystic and solid, and demonstrates contrast enhancement [4, 5]. Histologically, PAs are composed of neoplastic astrocytes with mild-to-moderate atypia and bipolar piloid processes. Most PAs demonstrate a biphasic pattern of growth with areas of increased cellularity and a dense fibrillary background alternating with hypocellular areas with microcysts; interspersed Rosenthal fibers and/or eosinophilic granular bodies (EGBs) are seen in classic cases most typically in the cerebellum, but are not a specific diagnostic feature. Areas of infiltration, indistinguishable from those of a diffuse astrocytoma, may be present at the periphery of the tumor [5].

PAs generally have an excellent prognosis and are classified as WHO grade I neoplasms [5]. Surgical excision alone is frequently curative, with a survival rate of greater than 95% at 10-year follow-up [2, 6]. This benign clinical course underscores the importance of accurately distinguishing these tumors from other glial neoplasms. Occasionally, the histological diagnosis can be problematic because PAs can demonstrate mitoses, foci of increased proliferative labeling index by immunohistochemistry for MIB1, microvascular proliferation and sometimes geographic necrosis, all features that are also encountered in high-grade gliomas [5]. At the same time, midline glioma with *H3 K27 M* mutation, a WHO grade IV tumor with dismal prognosis, can present with low-grade histology, especially on biopsy specimens [7]. Most PAs have activation of mitogen activating protein kinase (MAPK) signaling through numerous described alterations, the most common being a tandem duplication at 7q34 that results in *KIAA1459-BRAF* fusion, therefore demonstration of a known alteration is helpful in supporting a diagnosis of PA [8–10].

In this manuscript we describe a tumor with a novel fusion of *BRAF* with *GIT2*, hypothesized to result in activation of MAPK in a similar fashion as in PAs with the canonical *KIAA1459-BRAF* fusion.

## Case presentation

### Clinical history

**Fig. 1:**
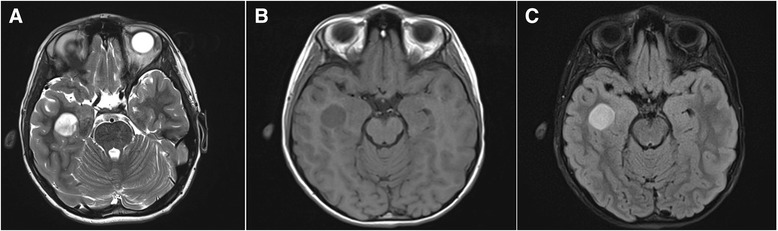
**a** MRI without contrast, axial section: a T2 hyperintense relatively well defined tumor in the right temporal lobe. **b** T1 sequence, slightly hyperintense well defined tumor in the right temporal lobe. **c** FLAIR sequence highlights the tumor; peritumoral edema is not seen

We report the case of a 10-year-old male patient with a 1-year-history of headaches that were thought initially to represent migraines. However, he progressed to having blurred vision and complex partial seizures characterized by jaw and mouth movements, teeth grinding and upper extremity stiffness/shaking. Magnetic resonance imaging without contrast showed a 2.0 × 1.9 × 1.8 cm tumor in the right temporal lobe along the inferolateral margin of the temporal horn of the right ventricle, with mild mass effect (Fig. 1a). The mass was slightly heterogeneous in signal characteristics, slightly hyperintense on T1 sequence (Fig. 1b) and peritumoral edema was not observed (Fig. 1c, FLAIR sequence). The patient underwent surgery and a gross total resection was achieved. A gray-tan, soft, partially gelatinous tumor was received by pathology.

### Histological and immunohistochemical stains

Hematoxylin-eosin-stained sections were performed on 4-μm thick formalin-fixed paraffin-embedded sections. Antibodies against glial fibrillary acidic protein (GFAP, Leica Biosystems, Richmond IL, clone GA5, predilute), NeuN (Chemicon, MilliporeSigma, Temecula, CA, clone A60, 1:100), IDH1(R132H) mutant protein (Dianova GmbH, Hamburg, Germany, Clone H09 1:25), OLIG2 (Cell Marque, Sigma Aldrich, Rocklin, CA, clone EP112, predilute), Ki67 (Cell Marque, Sigma Aldrich, Rocklin, CA, clone SP6, predilute) and BRAF V600E (Ventana, Tucson, AZ, clone VE1, predilute) were applied. Coverslips were mounted with Permount (Fisher Scientific). The slides were examined under an Olympus BX41 light microscope. Photographs were taken using an Olympus DP25 camera.

### Next generation sequencing

Next generation sequencing was performed using a previously described method [11]. In summary, DNA was analyzed by massively parallel sequencing using a solution-phase Agilent SureSelect hybrid capture kit. Somatic mutations in tumor DNA were detected using the exome-sequencing platform OncoPanel (Illumina HiSeq) in a CLIA-certified laboratory. Common single nucleotide polymorphisms (SNP) were accounted for using the following informatic steps: any SNP present at >0.1% in Exome Variant Server (NHLBI GO Exome Sequencing Project [ESP], Seattle, WA; URL: http://evs.gs.washington.edu/EVS/) or present in dbSNP was filtered. Variants also present in the COSMIC mutation database were rescued for manual review. Structural variants were detected using the BreaKmer algorithm, which identifies rearrangements and nucleotide-level breakpoints by realigning variant contigs generated from assembling all misaligned reads within the targeted NGS data [12].

## Results

**Fig. 2:**
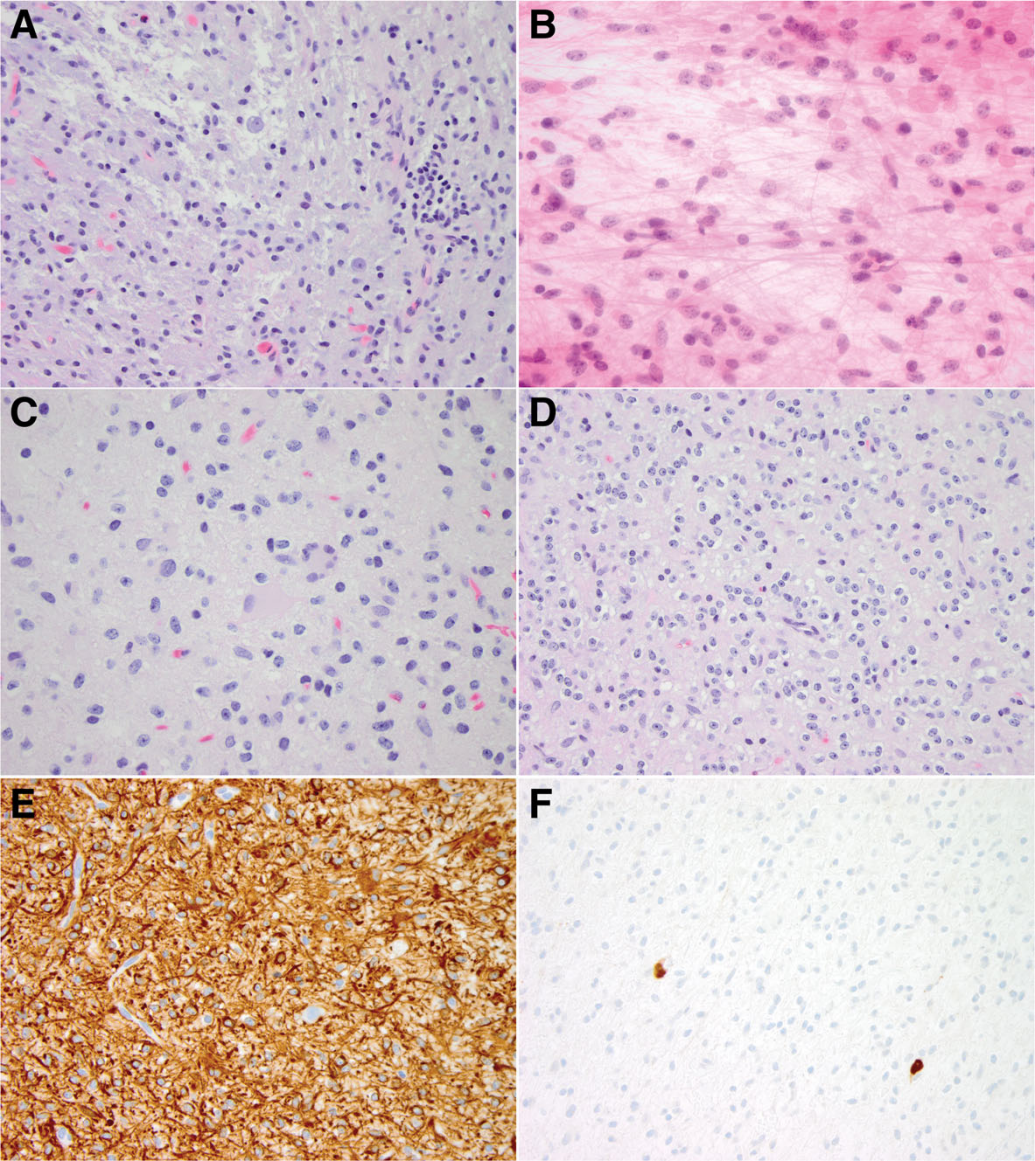
**a** H&E section showing some peripheral infiltration in this astrocytoma with mild nuclear atypia. **b** Smear preparation during the intraoperative microscopic examination, showing mildly atypical astrocytes with prominent bipolar piloid processes. **c** Dense, relatively hypercellular area with mild nuclear atypia and occasional multinucleated cells with peripheral nuclei (“penny on a plate”). **d** Focal oligodendroglioma-like morphology, with perinuclear clearing and round nuclei with speckled chromatin. **e** Extensive cytoplasmic GFAP immunostaining. **f** Low MIB (KI67) proliferation labeling index of 1–2%

H&E sections demonstrated a focally infiltrative tumor composed of mildly atypical cells (Fig. 2a) with prominent bipolar piloid processes (Fig. 2b). Areas of increased cellularity alternated with areas less cellular (Fig. 2a) and occasional multinucleated cells with eccentric nuclei (“pennies on a plate”) (Fig. 2c) were present. Some areas demonstrated oligodendroglioma-like morphology (Fig. 2d). Mitoses were difficult to find and there was no necrosis or microvascular proliferation. Unequivocal Rosenthal fibers or eosinophilic granular bodies were not identified. The tumor demonstrated extensive immunostaining with GFAP (Fig. 2e) and OLIG2 (not shown), and was negative for BRAF V600E and for IDH1 R132H mutant protein. A MIB-1 immunostain showed a proliferative labeling index of 1–2% (Fig. 2f). The tumor was provisionally signed out as low-grade astrocytoma with piloid features, W.H.O. grade I or II.

**Fig. 3:**
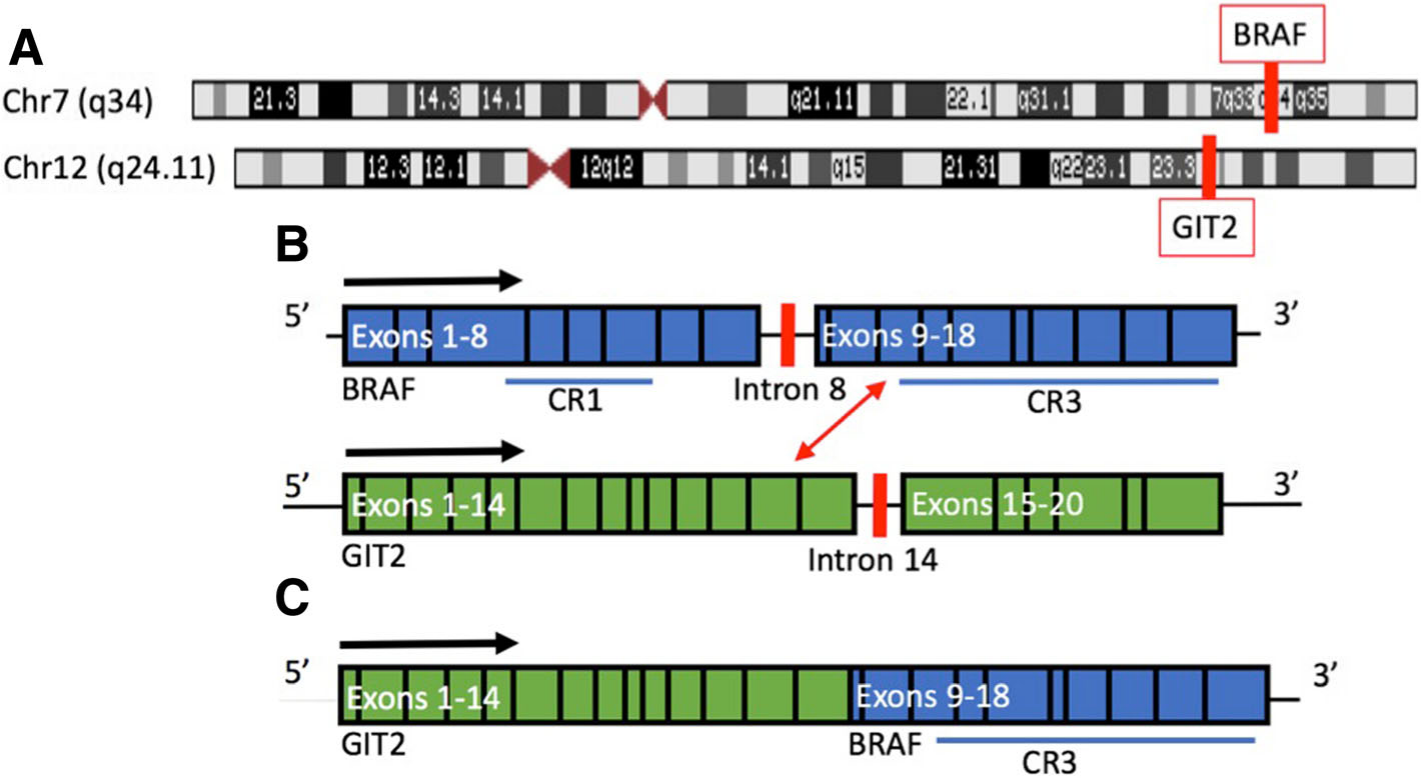
**a** Diagram of chromosomes 7 and 12 illustrating chromosomal breakpoints at location of BRAF (q34) and GIT2 (q24.11), respectively. **b** Schematic of BRAF gene, including autoregulatory domain (CR1) and kinase domain (CR3), and GIT2 gene. Breakpoints at intron 8 of BRAF and intron 14 of GIT2 are diagramed. **c** Resulting GIT2-BRAF fusion gene, with deletion of autoregulatory domain of BRAF. The kinase domain (CR3) is hypothesized to be constitutively activated in the resulting fusion protein (Additional file 1: Figure S1)

Molecular analysis using Oncopanel was performed, and the BreaKmer algorithm demonstrated a fusion involving intron 14 of *GIT2* and intron 8 of *BRAF*, resulting in loss of *BRAF* exons 1–8 (Fig. 3a-c). Overall, 43 split reads were detected out of 259 total reads demonstrating a translocation at base 110,387,138 of chromosome 12 within intron 14 of GIT2, and 140,494,011 of chromosome 7 within intron 8 of BRAF. 29 discordant reads were also detected (Fig. 3d). There were no copy number changes besides low copy gain of *SOX2* (3q26.33). There were no significant single nucleotide variants identified. Given the molecular test results, an integrative diagnosis of “low-grade astrocytoma with piloid feature most consistent with pilocytic astrocytoma, WHO grade I” was rendered.

### Follow-up

Given that the patient underwent a gross total resection, the patient is followed periodically with imaging studies and neuro-oncology consultation; radiotherapy or chemotherapy was not used. At nine months from surgery, the patient is stable, without evidence of recurrence.

## Discussion

PAs demonstrate MAPK signaling pathway activation through different genetic alterations. The first described and most common genetic event, *KIAA1549-BRAF* fusion, was described in 2008 by Bar et al. [8], who observed frequent gains at 7q34 and increased signaling in *BRAF-MEK-ERK* in PAs. More recent studies [9, 13, 14] describe numerous other genetic events that result in MAPK pathway activation: *BRAF V600E* mutations, *BRAF* intragenic deletions and insertions, *NTRK2* fusions, *FGFR1* and *KRAS* point mutations. Recently described *BRAF* fusion partners are *FAM131B, RNF130, CLCN6, MKRN1, and GNAII*. We report a novel inframe *GIT2-BRAF* fusion with breakpoints at intron 14 of *GIT2* (band q24.11) and intron 8 of *BRAF* (band q34), with retention of exon 9 and higher in *BRAF*. There were no significant copy number changes observed, and no single copy gain at 7q34, therefore this fusion is the result of a deletion, and not tandem duplication; the same mechanism of MAPK activation has been described in pilocytic astrocytomas with *FAM131B-BRAF* fusions. Because the deleted region of *BRAF* is identical to the one affected in other described fusions, resulting in deletion of the inhibitory domain of the *BRAF* and constitutive activation, most likely this fusion activates the MAPK signaling pathway.

*GIT2* encodes the ARF GTPase-activating protein GIT2, which plays a role in the regulation of cell-to-cell adhesion and cell motility. While overexpression of GIT2 has been described in the literature in breast and lung carcinomas [15, 16], this fusion with *BRAF* is novel in tumorigenesis and has not been described in any other type of tumor, hence it is difficult to predict the effect that the deletion will have on the *GIT2* gene. Although the glioma described in this case report had focal infiltration, the histologic features were widely within the range seen in other pilocytic astrocytomas reviewed at our institution and reported in the literature; there were no specific histologic features that could be directly associated to *BRAF-GIT2* fusion.

In the daily practice of neuropathology, it may be difficult to decide if the case presented is histologically WHO grade I or II due to its focally infiltrative nature. While areas of focal infiltration can be concerning for a histologic grade II neoplasm, many pediatric low-grade astrocytomas and glioneuronal tumors that are WHO grade I, including PA, have areas of infiltration. This is a feature seen in practice and mentioned in the literature, including in the revised WHO Classification of the Tumors of the Central Nervous System [5]. Such difficulty in precise histologic grading underscores the value of molecular findings in helping to predict clinical behavior. While there are infiltrative gliomas with MAPK pathway activation that may demonstrate progression over time, or have a first presentation with high-grade histology, these tumors usually have MAPK pathway activation through structural alterations in *FGFR* or *NTRK* family genes, or have pathogenic variants of *NF1* or *BRAF* [17–22]. Alternatively, structural rearrangements including fusions, duplications, and intragenic deletions involving the inhibitory domain of *BRAF* and thought to result in constitutive activation of the kinase domain, as seen in this case report, are very common in PA and possibly even specific to this neoplasm. They are associated with the increased survival expected in pilocytic astrocytomas and other WHO grade I pediatric gliomas, and therefore such a molecular finding may suggests a diagnosis of PA in the appropriate histologic context [23]. Although a body of literature suggests that a small percentage of diffuse astrocytomas, WHO grade II, have BRAF structural rearrangements, the progression to high-grade gliomas that is expected in diffuse astrocytomas is not expected in tumors with BRAF fusions [24].

## Conclusion

We are expanding the knowledge of genetic events that activate the MAPK signaling pathway in low-grade gliomas by describing a novel *GIT2-BRAF* fusion in PA. Given the evolving targeted therapeutic options for patients with recurrent or inoperable PAs, complete molecular characterization of these tumors is more important than ever.

## Additional file

Additional file 1: Figure S1. Representative image of BreaKmer interface illustrating GIT2-BRAF translocation. Sequenced contigs corresponding to BRAF are gray, with contiguous rainbow reads corresponding to bases that are part of GIT2. Sequence details of the translocation are shown at the bottom of the schematic (TIFF 25908 kb)

## Abbreviations

CLIA: Clinical Laboratory Improvement Amendments
EGB: Eosinophilic granular body
MAPK: Mitogen activated protein kinase signaling pathway
PA: Pilocytic astrocytoma.
SNP: Single-nucleotide polymorphism.
WHO: World Health Organization.
