# Supplementary figures and images for: A novel GIT2-BRAF fusion in pilocytic astrocytoma
Source: Diagn Pathol. 2017 Nov 15;12:82. doi: 10.1186/s13000-017-0669-5 (PMC5688665; doi:10.1186/s13000-017-0669-5)

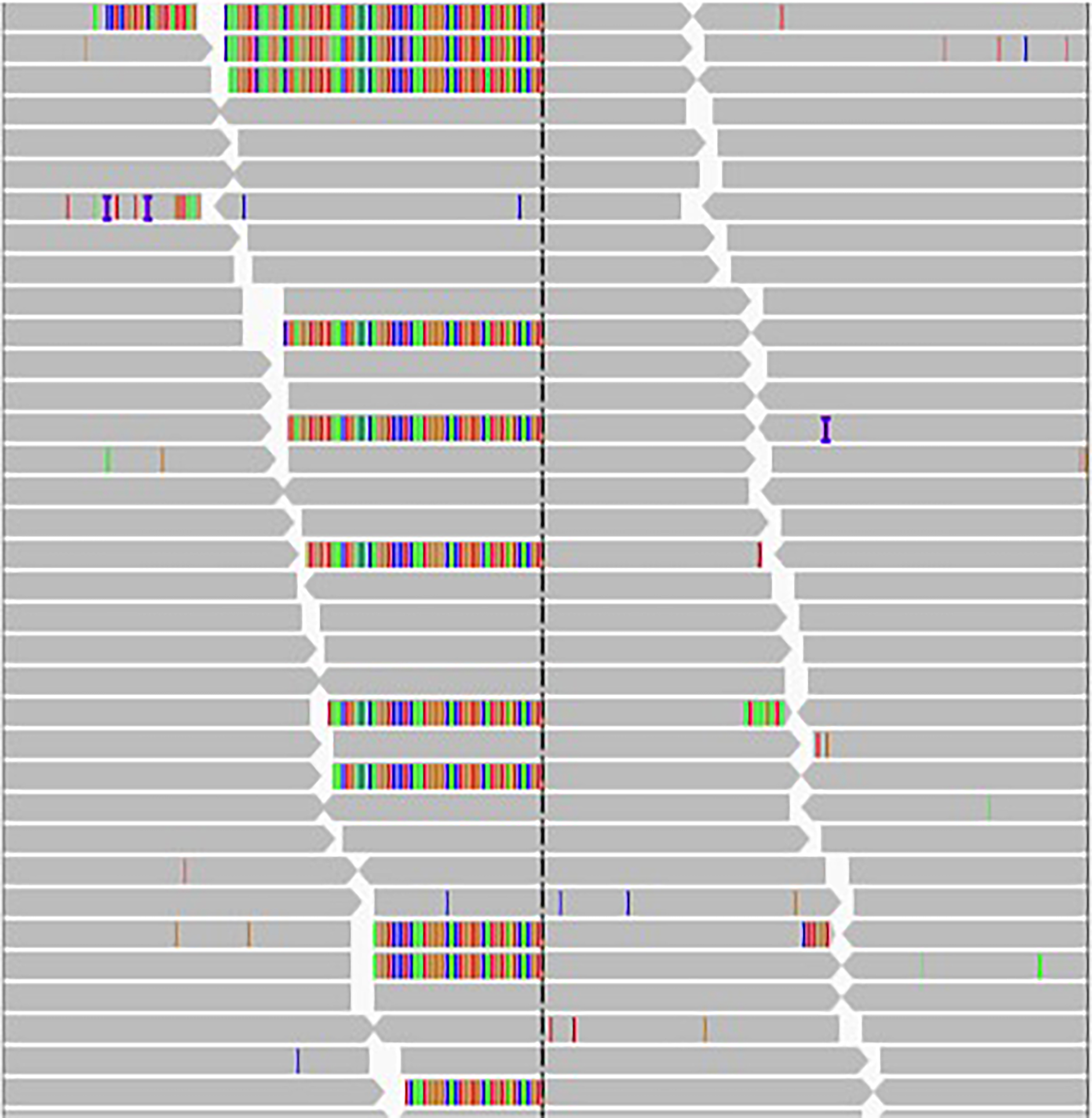

Supplement: Additional file 1: Figure S1. — Representative image of BreaKmer interface illustrating GIT2-BRAF translocation. Sequenced contigs corresponding to BRAF are gray, with contiguous rainbow reads corresponding to bases that are part of GIT2. Sequence details of the translocation are shown at the bottom of the schematic (TIFF 25908 kb) [file 13000_2017_669_MOESM1_ESM.tif]
